# Medical Society of Tennessee

**Published:** 1883-01-20

**Authors:** 


### *MEDICAL SOCIETY OF TENNESSEE—TRANSACTIONS.*

Transactions of the forty-ninth annual meeting of the Medical Society of the State of Tennessee, held at Memphis, May 9th 1882.

The following interesting papers are to be found in the Transactions, a neat publication of 186 octavo pages:

Address by the President, G. B. Thornton, M.D.

The Progress of State Medicine in Tennessee, by W. M. Clark, M.D.

Antisepsis, by Henry Ess, M.D.

Urethral Strictures, by W. F. Glenn, M.D.

The Principals Involved in the Management of Abortion, by Alex. Erskine, M.D.

Observations of the Five Yellow Fever Epidemics, occurring in the City of Memphis, Tenn., by D. D. Saunders, M.D.

Hypodermic Administration of Morphia in Convulsions of Children. by Thad. Donohue, M.D.

The Practical Bearings of Iridectomy, by A. G. Sinclair, M.D.

Psycological Medication, by G. A. Baxter, M.D.

Medical Education, by J. W. Davis, M.D.

A New Urethratome, by R. B. Nall, M D.

Typho-Malarial Fever, by T. K. Powell, M.D.

Reflex Troubles, the result of Rectal Irritation, and their Treatment, by W. F. Clary, M.D.

Epidemic Dysentery—as it manifested itself in Trenton, Tenn., and a portion of Gibson county, Tennessee, in the fall of 1880; its history and Etiology, by T. J. Happel, M.D.

The Prevention of the Puerperal Diseases, by Rich'd B. Maury, M.D.

Placenta Previa, by A. Jones, M.D.

### OFFICERS ELECT FOR 1883.

*President.*—W. F. Glenn, M.D., Nashville.

*Vice-Presidents*—For Middle Tennessee; W. F. Clary, M.D., Unionville. For West Tennessee: E. Miles Willett, M.D, Memphis. For East Tennessee: G. A. Baxter, M.D., Chattanooga.

*Secretary.*—C. C. Fite, M.D., Shelbyville.

*Treasurer.*—R Cheatham, M.D., Nashville.

